# Antithrombotic/Antiplatelet Treatment in Transcatheter Structural Cardiac Interventions—PFO/ASD/LAA Occluder and Interatrial Shunt Devices

**DOI:** 10.3389/fcvm.2019.00075

**Published:** 2019-06-07

**Authors:** Anna Olasinska-Wisniewska, Marek Grygier

**Affiliations:** Chair and 1st Department of Cardiology, Poznan University of Medical Sciences, Poznan, Poland

**Keywords:** antithrombotic, antiplatelet, ASD, PFO (patent foramen ovale), LAA (left atrial appendage) closure, Interatrial shunt device

## Abstract

Transcatheter interventions enable safe and efficient treatment of various structural heart diseases. However, therapy does not finished with the end of the procedure. Device thrombosis is a possible serious complication. Therefore, careful patient management should include optimal antiplatelet or antithrombotic medication to enhance safe and complete endothelial coverage of the implanted device. In case of thrombus formation careful diagnostic evaluation and prompt treatment is crucial. This paper provides an update to current knowledge and understanding of prevention and management of device related thrombosis.

## Patent Foramen Ovale (PFO)/atrial Septal Defect (ASD)

The percutaneous intervention has become a feasible and safe method of interatrial shunts closure.

According to current guidelines (1) percutaneous closure of ASD II is recommended in patients with significant shunt with signs of right ventricle overload and pulmonary vascular resistance (PVR) lower than 5 WU and should be considered in patients with paradoxical embolism. PFO closure may be proposed (2) to patients with a diagnosis of cryptogenic stroke after careful exclusion of other potential sources of embolism.

The procedure of ASD/PFO closure is commonly performed and relatively safe. However, thrombus formation is a recognizable and potentially harmful complication. This phenomenon is observed in 1.0–2.0% of patients (2) in all available types of devices. The risk of septal occluder device thrombosis may be even higher since asymptomatic patients may not undergo proper planned echocardiographic surveillance (3). Although uncommon, it may result in serious complications, including systemic embolism, and recurrent neurologic episodes (3). Thrombus formation may occur during procedure on either the delivery sheath or the device (4) or after the intervention, and has been noted even up to 5 years after the procedure (5). The incidence of thrombus formation is highest within the first 4 weeks after device implantation and is extremely rare after 8–12 months (6).

The majority of ASD/PFO occluders are composed of metal (usually nitinol) and a polyester mesh (4). Coagulation within the wire mesh facilitates fibrous tissue growth into the device which promotes a complete endothelialization and final occlusion of the shunt (7). Endothelialization and scar tissue formation on the implanted device is necessary to obtain complete closure of the intra-atrial communication and to prevent large subsequent thrombus formation (8). Increase in the coagulations cascade noted (9) after the procedure is an integral part of the sealing process (10). According to study by Anzai et al. (8) these processes may be visualized in transesophageal echocardiography (TEE) as plain forms of thrombi on the device and represent a natural healing process. Such findings do not necessarily suggest higher risk of embolization, and may resolve with time, with or without anticoagulation therapy.

Inclusion of pharmacological anticoagulation may counteract this desired process of healing and is associated with a risk for hemorrhagic complications. However, excessive coagulation may lead to uncontrolled thrombus formation on the device surface and the risk of embolic complications (7).

Currently preprocedural and postprocedural pharmacological therapy in the prevention of the device related thrombosis is commonly accepted and based on clinical practice and randomized clinical trials.

In majority of the centers aspirin (ASA) and clopidogrel are started preprocedurally, with a loading doses of 300 and 600 mg, respectively. Patients after ASD closure usually should receive dual antiplatelet therapy (DAPT) for 3 months and continue ASA up to 6 months. The decision to continue ASA therapy longer than 6 months is left at physician discretion.

Post procedural management after PFO closure is based on results of randomized clinical trials [RCT—CLOSE (11), CLOSURE I (12), DEFENSE-PFO (13), RESPECT Trail (14)]. Patients without history of thromboembolism, atrial fibrillation or other indications to use anticoagulation should receive DAPT therapy with aspirin and clopidogrel in the first six months and continue with a single drug for at least 5 years [based on latest consensus (2)]. The extension of the therapy with single antiplatelet drug beyond 5 years should be based on estimation of the balance between patient's overall risk of stroke for other causes and hemorrhagic risk. However, in the daily clinical practice DAPT is usually used for 6–8 weeks followed by single antiplatelet therapy up to 1 year (Supplementary Figure 1).

Sherman et al. (3) pointed out higher risk of thrombosis in atrial fibrillation and suggested reconsideration of the safety of the procedure and lifelong use of oral anticoagulation in this particular group of patients.

Nevertheless, device thrombosis was noted either in patients with antiplatelet therapy or anticoagulants. Nkomo et al. (15) described thrombus formation on CardioSEAL device despite warfarin use with acceptable international normalized ratio (INR). They explained that warfarin might reveal a procoagulant effect during initiation of therapy if the patient did not receive concomitant heparin at the same time.

Transthoracic echocardiography (TTE) is usually unable to detect thrombus formation on the device (16). Krumsdorf et al. (16) suggested a routine TEE examination in adult patients, including those with good echocardiographic windows, after 4 weeks, 6 and 12 months, however nowadays such practice is rather rare in majority of centers. For children, follow-up only by TTE as an imaging tool might be sufficient. Sherman et al. (3) proposed early (3 months) echocardiographic surveillance and TEE in cases when transthoracic echocardiography suggests thrombosis or when transthoracic images are suboptimal.

Once the thrombosis was identified, based on the literature, over 80% of patients were successfully treated with only conservative medical therapy (3, 8, 16). The mobility and potential friability of thrombus is associated with a high risk of embolization early after procedure and therefore requires an aggressive management (4, 17). Moreover, the observation that 76% of thrombi occurred on the left atrial side of a device highlights the risk of systemic embolization (3).

According to the literature reports thrombi were treated with UFH alone (18) or UFH with subsequent anticoagulation (16, 19), or only oral anticoagulant (8, 16), and even with recombinant tissue plasminogen activator with a glycoprotein IIb/IIIa inhibitor (tirofiban) (20) or a glycoprotein IIb/IIIa inhibitor (abciximab) with heparin (17). Summary of studies reporting DRT is presented in Supplementary Table 1.

The majority of thrombi resolves within 4 weeks to 6 months after introduction of anticoagulation (16). In analysis by Krumsdorf et al. (16) the thrombus disappeared after 4 weeks in 11 out of 17 patients. A TEE should be performed following anticoagulation treatment to confirm resolution of the device-related thrombus. Thrombolysis or even surgical removal of the device should be considered in patients who do not respond to medical therapy. In cases treated with surgery thrombus should be removed together with the device especially if unsuccessful medical treatment was associated with cerebral embolization (8, 16, 21, 22).

Several authors tried to discriminate predictors of device related thrombus formation after transcatheter closure. Sherman et al. (3) in their review pointed out that all types of commercially available devices had thrombosis reports. The data from their review in no way demonstrate that one device is superior to another. Krumsdorf et al. (16) reported 1,000 patients, 593 of them with PFO and 407 with an ASD. Twenty of them had device associated thrombi during the 1 or 6-month follow up. The thrombi occurred more often in the CardioSEAL (7.1%), the PFO- Star device (6.6%) or the StarFLEX (5.7%) devices compared to the Amplatzer device (0.0%) (*p* < 0.05). Paroxysmal atrial fibrillation after the procedure (6.2 vs. 20%; *p* < 0.05) and persisting atrial septal aneurysm despite effective transcatheter closure (1.3 vs. 20%, *p* < 0.01) were significant predictors for thrombus formation (16). A correlation with atrial septal aneurysm was however not mentioned in other reports. Thus, the significance of this finding should be treated with caution.

## Left Atrial Appendage Closure (LAAC)

Atrial fibrillation is the most common arthytmia and a second cause of stroke. The left atrial appendage (LAA) has a thrombogenic potential, particularly in the setting of atrial fibrillation (AF), and may be the main source (up to 90%) of the majority of AF-attributable emboli in patients with non-valvular atrial fibrillation. Percutaneous LAA closure was introduced either as an option to oral anticoagulants (USA) or to avoid long term oral anticoagulation in patients with contraindication to pharmacological therapy, including those with previous bleeding or at very high risk of bleeding complications (Europe) (23). Even 10–30% of patients may present relative, and 2–3% absolute contraindications to oral anticoagulants. The efficacy and safety of LAA occlusion in patients with AF has been demonstrated in several studies including two large randomized clinical trials the PROTECT-AF and PREVAIL and prospective registries (CAP, ASAP, EWOLUTION, ACP/Amulet European Registries) (24–30). Two major systems are currently used most frequently worldwide—Watchmann (Boston Scientific, USA) and Amplatzer Cardiac Plug/Amulet (Abbott, USA), and several other devices used occasionally or in clinical trials—Ultraseal (Cardia Inc, USA), WaveCrest (Coherex Medical, USA), Occlutech (Occlutech International AB, Sweden), LAmbre LAA Closure System (Lifetech, China).

Thrombus in the LAA confirmed by TEE usually precludes the LAAC, as it may be mobilized from the LAA during the procedure. If present, an attempt to resolve the thrombus should be first initiated—with 3–4 weeks of vitamin K antagonists (VKA) or novel oral anticoagulants (NOAC) until its complete resolution. There are only some exceptional reports of LAA closure despite presence of the thrombus (31).

It must be pointed out, that thrombus may occur within hours without anticoagulation in favorable circumstances (atrial fibrillation promotes prothrombotic activity in left atrium (LA) and LAA), thus periprocedural anticoagulation is necessary to prevent from intraprocedural thrombotic complications. Anticoagulants are usually stopped 1–3 days before the procedure and INR should be normalized if VKA is used, however some operators advocate to perform procedure on anticoagulants similarly to atrial fibrillation ablation. Patient receives aspirin (usually higher dose -300 mg) and clopidogrel 1 day before the procedure. Unfractionated heparin is given intraprocedurally (to achieve ACT 250–350 s) after atrial septum puncture. The use of brain protection devices during LAA procedures [like Sentinel, Boston Scientific (USA)] is under clinical evaluation (32).

The thrombus formation results from a local tissue response to an implantation and may occur on all available devices. The healing response to nitinol-based devices is initiated by thrombotic material formation and then its transformation into connective tissue within 4 weeks (33). Accumulation of plane thrombus may however turn into excessive, unwanted thrombus formation (33).

LAA closure enables discontinuation of long term anticoagulation. However, shortly after the procedure antiplatelet or antithrombotic prophylaxis is used to prevent DRT. Published data regarding DAPT or OAC choice after the procedure are conflicting. In the study of Chun et al. (34) DAPT administered for 6 weeks after LAAC was associated with less frequent device-related thrombus (1,7%) compared to OAC (15,8%) regardless of the type of device. Plicht et al. (35) reported a higher rate of devices related thrombus (DRT) (Amplazter Cardiac Plug) treated with DAPT. It is possible that nitinol cage (Watchman, Boston Scientific) and nitinol plug devices (Amplatzer, St Jude Medical) may have different thrombogenicity and endothelialization profiles and different needs for antithrombotic prevention. Despite several published analyses, the precise duration and type of optimal therapy is unclear. Current treatment strategies for oral anticoagulants and/or anti-platelet therapy have been derived largely from protocols of randomized clinical trials with Watchman device for LAAC (Supplementary Figure 1).

In fact, in patients eligible for oral anticoagulants, the scheme of post-implantation treatment is similar to PROTECT-AF one (27): warfarin with aspirin are administrated for 45 days, followed by DAPT—clopidogrel (75 mg daily) until the 6-month and aspirin (81–325 mg daily) for life. New studies suggest that use of NOAC instead of warfarin within the first 45 days after interventional LAA closure is safe and effective (36, 37).

Patients with contraindication for oral anticoagulants, who constitute majority of population qualified to LAA closure at least in Europe, receive DAPT as a reasonable alternative based on the large registries (38). The multicenter, prospective, nonrandomized ASAP study (28) was the first one conducted in patients who were considered ineligible for even short term treatment with warfarin. Use of antiplatelet agent (clopidogrel or ticlopidine) for 6 months and lifelong aspirin was proved to be safe alternative to OAC. Similarly, in the study by Urena et al. (39) antiplatelet therapy consisting of aspirin (80–325 mg/24 h) plus clopidogrel (75 mg/24 h), or aspirin or clopidogrel alone were administered according to the operators' discretion for 30–180 days after the AMPLATZER Cardiac Plug (ACP) implantation, and afterwards single- antiplatelet therapy was introduced. No cases of device thrombosis were observed in the 6-month follow-up. In EWOLUTION registry (40), following LAA closure with Watchman device, patients received DAPT, VKA, NOAC, single antiplatelet or no therapy (60.3, 15.4, 10.9, 7, and 6.5%, respectively). Device thrombus (2.6%) and stroke (0.4%) rates were low and did not vary among different post-implantation medication strategies (40).

The incidence of thrombus formation on devices varies widely in current registries, and ranges between 0 and 17.6% (33, 35, 41–46), with most of them reporting 2–4% (Supplementary Table 1). These differences may be explained by the different sample sizes, lack of consensus on the definition of device-associated thrombus, or reporting bias related to different imaging methods, the variations in imaging technique (TEE, computed tomography (CT), 4-dimensional CT) and frequency of imaging assessment during follow-up (41, 44). Moreover, in daily practice up to 72% of patients do not undergo LAA imaging during follow-up (except the first one after 6 weeks), which may be explained by the overall frailty of these high risk patients or a belief that the result of TEE would not change strategy in patients with high risk of bleeding.

Older age and history of ischemic stroke or transient ischemic attack (TIA), permanent atrial fibrillation, vascular disease, higher CHA_2_DS_2_-VASc, large LAA diameter and spontaneous echo contrast were risk factors for device-related thrombus, whereas DAPT and oral anticoagulants use at discharge were associated with a lower risk of thrombus formation (33, 35, 41, 44, 46, 47). Among procedural factors—larger device size and deep implantation—were related to the risk of thrombus formation (33, 47). Main et al. (24) found that the nidus for most DRT appeared to be the central portion of the device (“threaded insert”). Although the remainder of the LA facing portion of the device is covered by a permeable polyester fabric, the center portion contains exposed nickel titanium alloy (nitinol) (24). Newer generation device—Watchman FLX has minimal area of metal screw facing the left atrium to encourage endothelialization and reduce post-implant thrombus formation (48). Ketterer et al. (45) found a clopidogrel resistance documented by platelet function testing in 3 of 4 patients (75%) with DRT. They suggested platelet activity and response to clopidogrel testing and changing to an alternative P2Y12 receptor inhibitor as an option (45). Contrary, Plicht et al. (35) found no genetic risk for clopidogrel resistance or other coagulation disorders in patients with thrombi.

Device related thrombus may occur during the procedure (44, 49) and at every stage of follow up after the procedure (33). In the analysis of device arms of 4 prospective FDA trials (PROTECT-AF, PREVAIL, CAP and. CAP2) by Dukkipati et al. (44) device related thrombus was detected on 0.8% at 45 days and in 1.7% at 6 months and 1.8% at 12 months. According to Main et al. (24) incident DRT has higher prevalence at 6 and 12 months when compared with 1.5 months post-procedure. It may be partially explained with the hypothesis that DRT may initially be too thin to be detected by TEE or CCT and become apparent at later stages of follow-up (33).

Recently published papers showed that thrombus on the device is an independent factor strongly associated with strokes and TIA during follow-up (41, 42, 44). Considering all types of devices, a 4 to 7.3% rate of ischemic stroke or TIA has been reported in patients with device-related thrombus (41). It must be however emphasized that this population of patients has the propensity for other sources of emboli, including vascular disease.

In the largest prospective analysis of 1,739 patients in the PROTECT-AF and PREVAIL trials and two registries (CAP—Continued Access to PROTECT AF registry and CAP2—Continued Access to PREVAIL registry) (44) the incidence of device-related thrombus (DRT) was 3.7%. Twenty-five percent of patients with DRT experienced an ischemic stroke or systemic embolism in comparison with 6.8% patients without DRT which means that DRT 3-fold increased risk of stroke or systemic embolism. It should be however mentioned that the majority of patients with DRT (73.8%) did not experience any stroke/systemic embolism and the majority of strokes/systemic embolisms (86.6%) occurred in patients who never manifested a DRT (44).

There are conflicting data concerning risk of thrombus formation in patients with incomplete LAA occlusion. Hypothetically, incomplete sealing of the orifice may enhance thrombus formation and embolization of thrombi potentially leading to strokes. Lam et al. (50) described a case of thrombus formation in a patient with incomplete LAA closure with a Watchman device without clinical symptoms of embolization. Complete resolution of thrombus was achieved after 3 months of warfarin treatment and transcatheter LAA closure with a second-generation Amplatzer Cardiac Plug was performed. On the other side, Viles-Gonzalez et al. (51) performed a study to assess the risk of stroke related to incomplete occlusion and potential risk of thrombus formation. They did not observed any case of thrombus in patients with residual leak. According to their argumentation it could be postulated that even partial occlusion of the LAA could prevent large thrombus from migrating into the systemic circulation and that small clots may not have clinical significance.

Reddy et al. in PROTECT -AF trial (27) used follow-up TEE imaging performed at 45 days, 6 months, and 12 months to assess for device stability, peri-device leaks, and device-related thrombus. Dukkipati et al. (44) suggested a reevaluation of the TEE surveillance strategy and they proposed 1. routine additional TEE surveillance at 6 months 2. an escalated TEE monitoring strategy targeted to patients with thrombus risk factors such as permanent AF, lower left ventricle ejection fraction, history of stroke/TIA, vascular disease and larger LAA diameter, 3 delaying the first TEE to the 4 month time point instead of 6 weeks, because the thrombus occurs most likely after OAC discontinuation at 6 weeks and indeed after receiving DAPT. These are not a standard recommendation, however their adaptation seem to be reasonable in some particular groups of patients.

The manifestation of the DRT in fact impels the anticoagulation introduction—warfarin or “new” anticoagulants—rivaroxaban, dabigatran, apixaban (38, 44, 52). This treatment should be continued for weeks to months until thrombus resolution is confirmed by TEE. However, majority of patients qualified to LAA occlusion (at least in Europe) have by definition strict contraindication for treatment which enhances bleeding. Therefore, the long-term strategy remains uncertain and several factors, including patient characteristics (CHA_2_DS_2_-VASc score, HAS-BLED score, creatinine clearance), echocardiographic findings (low left ventricle ejection fraction, “smoke” in left atrium, decreased LAA velocity), and procedural factors (large device, deep implantation, incomplete closure) should be considered. After thrombus resolution a careful and individually driven surveillance is necessary, since thrombus may reappear and currently there is no established scheme for them.

## Interatrial Shunt Devices

Interatrial shunt devices were developed to reduce the left atrial pressure in patients with chronic heart failure with preserved EF (HFpEF) and with reduced EF (HFrEF) while avoiding excessive left to right shunting. Three devices—V-Wave (V-Wave, Caesarea, Israel), interatrial septal device system-IASD (DC Devices Inc, Tewksbury, MA, USA) and atrial flow regulator (AFR) (Occlutech, Sweden)—are currently available to create a restrictive interatrial communication (53).The last one is used also in clinical studies in patients with pulmonary hypertension resistant to pharmacological treatment.

The observational data are still limited. First, Søndergaar et al. (54) presented the results of pivotal trial with interatrial septal device system (IASD), implanted in eleven patients with HFpEF. They did not observe any patient with device thrombosis and suggested that with the particular construction of the device, mainly the flat device legs and used pharmacological protocol the risk of stroke would be low. In the REDUCE LAP-HF I study (55) one of 22 patients of a treatment group had a small thrombus which was observed on the tip of the device delivery system in the right atrium. The delivery system was removed and exchanged. A new system was then reinserted, and the IASD device was successfully implanted. The one month follow up of a study group was uncomplicated. There are however no strict data on echocardiographic management in the follow up.

The V-Wave shunt device is another shunt prosthesis which first-in-man implantation was described in 2015 by Amat-Santos et al. (56). Del Trigo et al. (57) presented first results in 10 patients and then Rodes-Cabau et al. (53) expanded the study group to the overall 38 patients with HFrEF and HFpEF. The authors also did not observe thrombus formation during and after the V-Wave device implantation.

Further studies are needed to determine the optimum pharmacological treatment after shunt device implantation (57). Treatment with oral anticoagulation for 3 months followed by ASA monotherapy lifelong was used after V-Wave implantation (53, 58). In patients after IASD implantation aspirin with clopidogrel for 6 months followed by ASA monotherapy lifelong were implemented and in those with indications for oral anticoagulation and/or antiplatelet therapy for a co-existing condition the preprocedural regimen was continued (55, 58). However, this frail population should be also assessed in terms of increased risk of bleeding during antithrombotic and antiplatelet treatment (58).

## Summary

Device related thrombosis after percutaneous procedures is rare but can be associated with serious complications. Therefore, optimal antiplatelet/antithrombotic treatment as well as careful echocardiographic assessment in follow up must be implemented in those patients. When detected, device related thrombosis warrants treatment with anticoagulation and aggressive follow-up with appropriate surveillance imaging.

## Author Contributions

AO-W and MG contributed substantial contributions to the conception or design of the work; or the acquisition, analysis or interpretation of data for the work. AO-W and MG drafted the work and revised it critically for important intellectual content, provided approval for publication of the content. AO-W and MG both agree to be accountable for all aspects of the work in ensuring that questions related to the accuracy or integrity of any part of the work are appropriately investigated and resolved.

### Conflict of Interest Statement

The authors declare that the research was conducted in the absence of any commercial or financial relationships that could be construed as a potential conflict of interest.
