# Supplementary material for: Antithrombotic/Antiplatelet Treatment in Transcatheter Structural Cardiac Interventions—PFO/ASD/LAA Occluder and Interatrial Shunt Devices
Source: Front Cardiovasc Med. 2019 Jun 7;6:75. doi: 10.3389/fcvm.2019.00075 (PMC6568033; doi:10.3389/fcvm.2019.00075)
Supplement: Supplementary file 1 [file Table_1.pdf]

Study	Procedure/device	No of patients (all)	No of patients with DRT or case report	DRT signs and/or diagnostics	Treatment of DRT
Yorgun H et al. 2011 [4]	ASD closure / Amplatzer septal occluder	2	Case report	Acute thrombus periprocedurally	heparin iv and warfarin at discharge
Raghu A et al. 2017 [5]	ASD closure / Amplatzer septal occluder	29-year old female	Case report	Multiple cerebral, cerebellar and brain stem emboli	Warfarin
Kovacevic P et al. 2017 [6]	ASD closure / CardioSEAL-STARFlex and Amplatzer occluder	2	1 patient with DRT	Embolisation, massive pulmonary embolism	surgery, death (related to right ventricle failure;
Anzai H et al. 2004 [8]	ASD or PFO closure / CardioSEAL and Amplatzer septal occluder	66, but 50 had TEE in follow up	5 (all related to CardioSEAL device)	No thromboembolic events	Warfarin,<br>1 patient - surgery
Nkomo VT et al. 2001 [15]	PFO closure / CardioSEAL	2	Case reports	Intermittent palpitations, headaches, photophobia Transient episodes of blurrred vision, transient episode of global amnesia, TEE	1:Surgery,<br>2: iv heparin, warfarin, surgery
Krumsdorf U et al. 2004 [16]	ASD and PFO closure/ Rashkind, Buttoned,ASDOS, Angel Wings, cardioSEAL, StarFLEX, Amplatzer, Helex, PFO-Star	1000	5 ASD and 15 PFO	3 minor strokes, 1 TIA	1 heparin,<br>12 warfarin<br>4 heparin and warfarin<br>3 surgery (1 after unsuccessful fibrynolysis; 1 after long term warfarin; 1 sent to surgery without pre-treatment)
Willcoxson FE et al. 2004 [17]	ASD closure / Amplatzer septal occluder	12-year old male	Case report	Intraprocedural thrombus formation	Abciximab and heparin, discharged with ASA and clopidogrel
Acar P et al. 2002 [18]	ASD closure / Amplatzer septal occuder	50-year-old female	Case report	Intraprocedurally	Heparin
Chessa M et al. 2002 [19]	ASD closure / CardioSEAL/STARFlex and Amplatzer septal occluder	417	1 patient	immediately after the device delivery	Retrieval with a basket catheter, anticoagulant therapy for 6 months

Vanderheyden M et al. 2002 [20]	PFO closure / PFO Starflex	46-year old female	Case report	No symptoms, routine TEE	Thrombolysis (r-tpa) and tirofiban, then warfarin and clopidogrel
Uysal F et al. 2016 [21]	ASD closure / Amplatzer septal occluder	One 17-year-old patient	Case report	Symptoms not reported, TEE	Surgery
Divchev D et al. 2007 [22]	ASD closure/ StarFLEX	69-year-old female	Case report	No symptoms, planned TEE	Surgery
Main ML et al. 2016 [24]	LAAC / Watchman	485	27	Planned TEE<br>Stroke, death	Not reported
Pracon R et al. 2018 [33]	LAAC / Watchman and Amplatzer Cardiac Plug/Amulet	102	7	1 stroke	ASA+LMWH<br>DAPT+LMWH<br>LMWH/OAC+DAPT<br>ASA+clopidogrel
Chun KRJ et al. 2013 [34]	LAAC / Watchman and Amplatzer Cardiac Plug	80	4	Planned TEE	Patients on DAPT - additional subcutaneous enoxaparin<br>Patients on OAC - intensified phenprocoumon
Plicht B et al. 2013 [35]	LAAC / Amplatzer Cardiac Plug	34	6	Planned TEE<br>No strokes occured	Iv heparin or VKA
Fauchier L et l. 2018 [41]	LAAC / Watchman and Amplatzer Cardiac Plug and Amplatzer Amulet	469	26	4 strokes<br>Planned TEE and CT	OAC
Lempereur M et al. 2017 – review [42]	LAAC / Watchman and Amplatzer Cardiac Plug and Amplatzer Amulet	Review of 30 studies 2118	82	TEE<br>Neurological events – TIA (2) and strokes (4)	LMWH<br>OAC<br>DAPT, SAPT<br>Unknown
Dukkipati SR et al. 2018 [44]	LAAC / Watchman	1739	65	16 patients had stroke or systemic embolism	Not reported
Ketterer U et al. 2016 [45]	LAAC / Watchman	46	4	Planned TEE	Phenprocoumon in 1 patient<br>Apixaban in 3 patients
Sedaghat A et al. 2017 [46]	LAAC / Amplatzer Amulet Device	24	4	TEE<br>1 TIA	Apixaban, edoxaban, dabigatran

Kaneko H et al. 2017 [47]	LAAC / Watchman	78	4	Planned TEE	Not reported
Lam SC et al. 2015 [50]	LAAC / Watchman	1	Case report	TEE in observation	Warfarin and subsequent second device (Amplatzer Cardiac Plug) implantation
Qazi AH et al. 2016 [52]	LAAC / Watchman	1	Case report	TEE in observation	DRT resolution on dabigatran, recurrence of DRT after completing dabigatran, introduction of rivaroxaban
Feldman T et al. 2018 REDUCE LAP-HF I [54]	Transcatheter Interatrial Shunt Device	94	1	Intraprocedurally – thrombus on the tip of the delivery system	Delivery system was removed and exchanged

Table 1. Summary of published reports of thrombus formation
