# Supplementary material for: Antithrombotic/Antiplatelet Treatment in Transcatheter Structural Cardiac Interventions—PFO/ASD/LAA Occluder and Interatrial Shunt Devices
Source: Front Cardiovasc Med. 2019 Jun 7;6:75. doi: 10.3389/fcvm.2019.00075 (PMC6568033; doi:10.3389/fcvm.2019.00075)
Supplement: Supplementary file 2 [file Image_1.pdf]

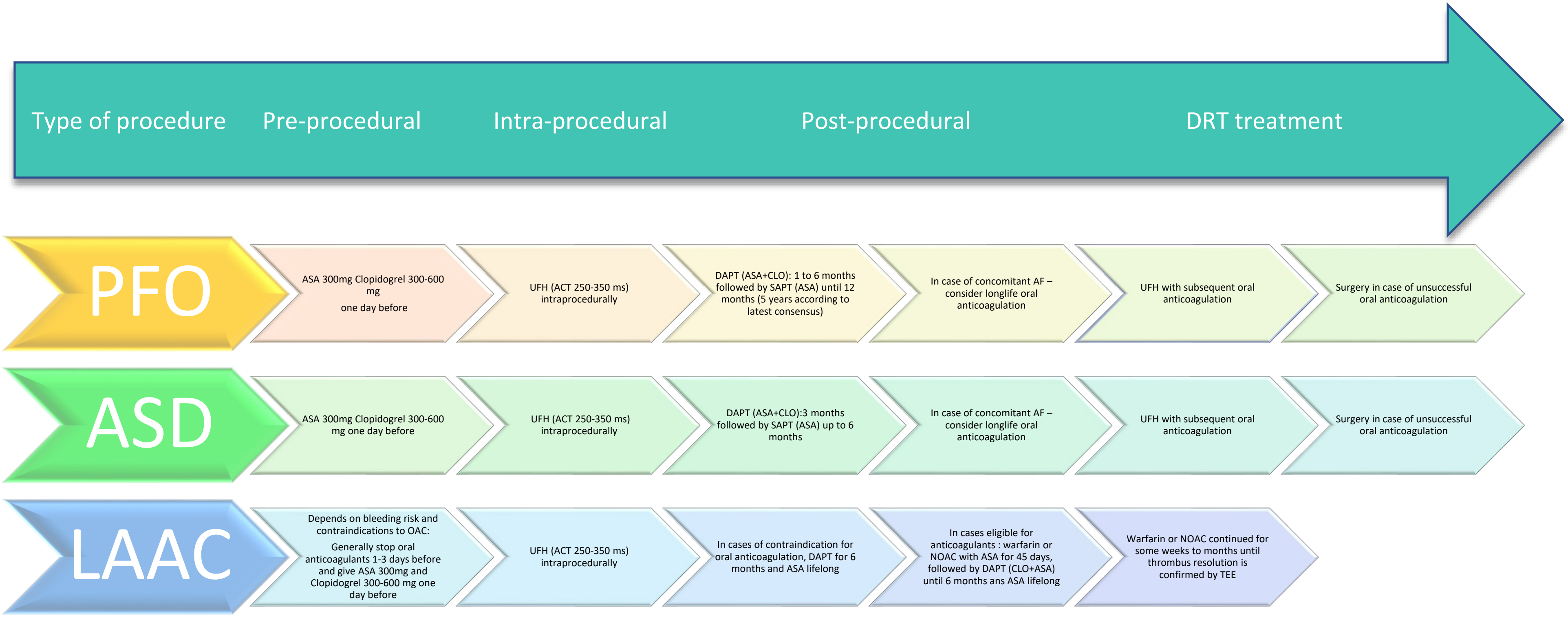


PFO/ASD persistent foramen ovale, atrial septal defect, LAAC left atrial appendage closure, DRT – device related thrombus, UFH unfractionated heparin, DAPT dual antiplatelet therapy, OAC – oral anticoagulants, NOAC novel oral anticoagulants, ASA – aspirin, CLO clopidogrel, TEE transoesophageal echocardiography

**Figure 1**. Treatment protocol in structural cardiac interventions
